# Comparative transcriptomic and metabolic analysis of wild and domesticated wheat genotypes reveals differences in chemical and physical defense responses against aphids

**DOI:** 10.1186/s12870-019-2214-z

**Published:** 2020-01-13

**Authors:** Zhaniya S. Batyrshina, Beery Yaakov, Reut Shavit, Anuradha Singh, Vered Tzin

**Affiliations:** 1 French Associates Institute for Agriculture and Biotechnology of Drylands, Jacob Blaustein Institutes for Desert Research, Ben-Gurion University of the Negev, Sede Boqer Campus, 8499000 Midreseht Ben Gurion, Beer-Sheva Israel; 2 Ilse Katz Institute for Nanoscale Science and Technology, Ben-Gurion University of the Negev, Beer-Sheva, Israel

**Keywords:** Aphid infestation, Benzoxazinoids, Defense, Domestication, *Rhopalosiphum padi*, Trichome, *Triticum aestivum*, *Triticum turgidum*, Wheat

## Abstract

**Background:**

Young wheat plants are continuously exposed to herbivorous insect attack. To reduce insect damage and maintain their growth, plants evolved different defense mechanisms, including the biosynthesis of deterrent compounds named benzoxazinoids, and/or trichome formation that provides physical barriers. It is unclear whether both of these mechanisms are equally critical in providing an efficient defense for wheat seedlings against aphids—an economically costly pest in cereal production.

**Results:**

In this study, we compared the transcriptome, metabolome, benzoxazinoids, and trichome density of three selected wheat genotypes, with a focus on differences related to defense mechanisms. We chose diverse wheat genotypes: two tetraploid wheat genotypes, domesticated durum ‘Svevo’ and wild emmer ‘Zavitan,’ and one hexaploid bread wheat, ‘Chinese Spring.’ The full transcriptomic analysis revealed a major difference between the three genotypes, while the clustering of significantly different genes suggested a higher similarity between the two domesticated wheats than between either and the wild wheat. A pathway enrichment analysis indicated that the genes associated with primary metabolism, as well as the pathways associated with defense such as phytohormones and specialized metabolites, were different between the three genotypes. Measurement of benzoxazinoid levels at the three time points (11, 15, and 18 days after germination) revealed high levels in the two domesticated genotypes, while in wild emmer wheat, they were below detection level. In contrast to the benzoxazinoid levels, the trichome density was dramatically higher in the wild emmer than in the domesticated wheat. Lastly, we tested the bird cherry-oat aphid’s (*Rhopalosiphum padi*) performance and found that Chinese Spring is more resistant than the tetraploid genotypes.

**Conclusions:**

Our results show that benzoxazinoids play a more significant defensive role than trichomes. Differences between the abundance of defense mechanisms in the wild and domesticated plants were observed in which wild emmer possesses high physical defenses while the domesticated wheat genotypes have high chemical defenses. These findings provide new insights into the defense adaptations of wheat plants against aphids.

## Background

Crop plants are continually exposed to different environmental stress conditions, such as herbivore infestation [1]. Insect herbivory is a crucial factor in yield loss and quality degradation in agricultural crop production. Average losses can reach 20–30% of yield, and in some cases, they can cause a total yield loss [2]. One of the most economically significant herbivores is found in the aphid family (Hemiptera: Aphididae) [3], a piercing-sucking pest that feeds on the phloem sap. Aphid infestation causes direct damage by consumption of water and nutrients, as well as indirect damage by plant virus transmission [4–6]. In response to insect infestation, plants produce constitutive and inducible defenses to reduce damage and enhance their own fitness [7]. Although many plant defenses are produced constitutively during a specific developmental stage, regardless of insect attack, others are inducible in response to insect damage. Examples of herbivore-induced defense mechanisms are the accumulation of toxic chemicals such as benzoxazinoids, glucosinolates, and alkaloids, which are classes of specialized metabolites that function as deterrents. Another mechanism is mechanical defense, including physical barriers such as the increased density of thorns, spikes, or trichomes [8–13]. Most of the toxic defenses are abundant in young seedlings and decrease during development toward the juvenile stage [14–16]. The herbivore-induced mechanisms are mediated by the modification of signaling (i.e., jasmonic and salicylic acid) [17], which allows the plants to conserve metabolic resources and energy to be directed toward growth and reproduction in the absence of insect herbivory.

Wheat is a staple crop that provides 20% of the world population’s caloric and protein intake [18]. It was first domesticated more than 10,000 years ago, making it one of the earliest domesticated crops [19]. The process of domestication, centuries of cultivation, and modern wheat breeding have led to the reduction or narrowing of genetic variation compared to their wild ancestors [20]. This reduction is due to the small initial crop population, coupled with intense selection for agronomic traits, considered as a “domestication bottleneck” [21, 22]. Adaptation of domesticated wheat and wheat varieties to local conditions intensified the reduction, giving rise to landrace cultivars [23]. Moreover, the cultivation of a germplasm with a narrow genetic base entails a risk due to genetic vulnerability to biotic and abiotic stresses, possibly resulting in severe crop losses. By using wild relatives of wheat as a proxy, the genetic diversity of agriculturally important traits can be contrasted before and after domestication [21]. It has been suggested that several agriculturally important traits, such as biotic and abiotic stress resistance, significantly decreased during wheat domestication [24, 25]. This lowered resistance, reported for responses to herbivore attack [26, 27], bacterial blight [28], and fungal disease [29], was revealed in domesticated members of the plant families Fabaceae and Brassicaceae [25]. In contrast, increased resistance of domesticated wheat to aphids was also reported [30]. It was suggested that the tuning of plant domestication defense mechanisms is dependent on pest feeding habits [31].

Integration between transcriptomic and metabolic datasets is commonly used to expose how insects may modify their host plant to their advantage [32, 33]. In this research, we investigated the differences in the transcriptome and metabolome of wheat seedlings, and we studied the effect on the chemical and physical defenses against *Rhopalosiphum padi* aphids. The variation between *Triticum* species for cereal aphid resistance has demonstrated the potential of using tetraploid wheat to reveal plant defense mechanisms [34–36]. Therefore, we focused our research on three representative wheat genotypes: i) Svevo, a tetraploid durum wheat cultivar (*Triticum turgidum ssp. durum*), ii) a wild emmer, Zavitan, a tetraploid ancestor of modern domesticated tetraploids (*Triticum turgidum ssp. dicoccoides*); both tetraploids have been intensively investigated as potential sources for resistance genes and markers [30, 37]; and iii) the spring wheat genotype Chinese Spring, which is widely used as a reference genome and for cytogenetic studies [20, 38–43]. We investigated young wheat seedlings (11–18 days old), which produce high levels of chemical and physical defenses. The chemical deterrent metabolites (benzoxazinoids) and the physical defenses (trichomes) were analyzed and compared with *R. padi* aphid reproduction. Altogether, this study evaluates the dynamics of defense mechanisms in response to aphid attack.

## Results

### Overview of the transcriptomic dataset differences between the three wheat genotypes

**Fig. 1:**
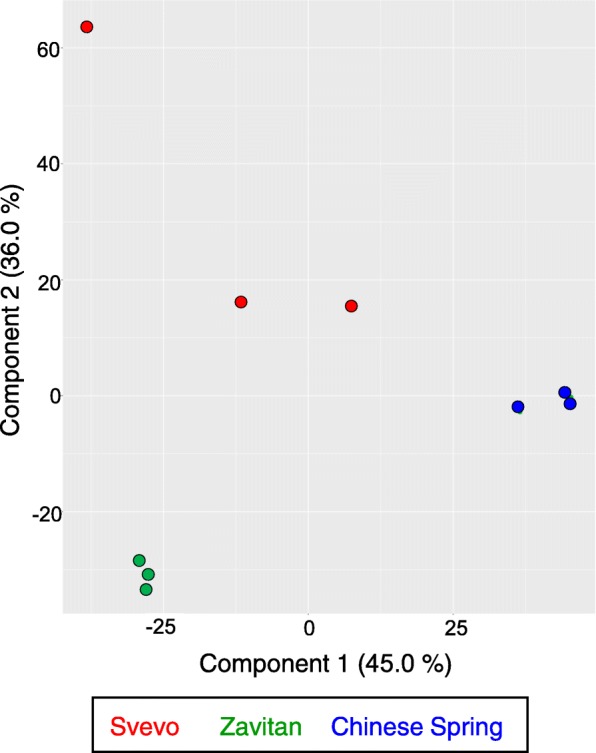
A principal component analysis (PCA) of the three wheat transcriptomic datasets. The PCA plot comprises 42,474 genes identified by transcript profiling (RNA-seq) of the three wheat genotypes, Svevo, Zavitan, and Chinese Spring, as 11-day-old seedlings (*n* = 3)

To investigate the global transcriptomic profile of the three wheat genotypes, leaves from 11-day-old seedlings were collected from genotypes with the same phenology (two-leaf stage; Additional file 2: Figure S1). A comparison of transcriptomic data was performed with annotated gene models found in the Chinese Spring reference genome sequence [20, 38], and the mapped sequence reads showed high similarity between the genotypes: Svevo, 95.85%; Zavitan, 94.68%; and Chinese Spring, 96.72% from the total mapped reads (Additional file 1: Table S1). Due to differences in the number of subgenomes, we eliminated all the transcripts that were annotated to the D subgenome or an unidentified subgenome (U). This analysis revealed 42,474 transcripts (Additional file 1: Table S2). The total transcript levels were used to conduct a principal component analysis (PCA) plot. As presented in Fig. 1, the PCA plot indicated that samples from each genotype were clustered with one another, while the genotypes were totally separated from each other, with component 1 (45%) showing a separation of Chinese Spring from the tetraploid wheat genotypes, and component 2 (36%) separating the wild (Zavitan) and cultivated (Svevo and Chinese Spring) wheat genotypes. Overall, the transcriptomic analysis of the three genotypes showed a unique pattern for each one.

### Clustering of expression patterns and pathway enrichment and gene ontology in the transcriptomic data

**Fig. 2:**
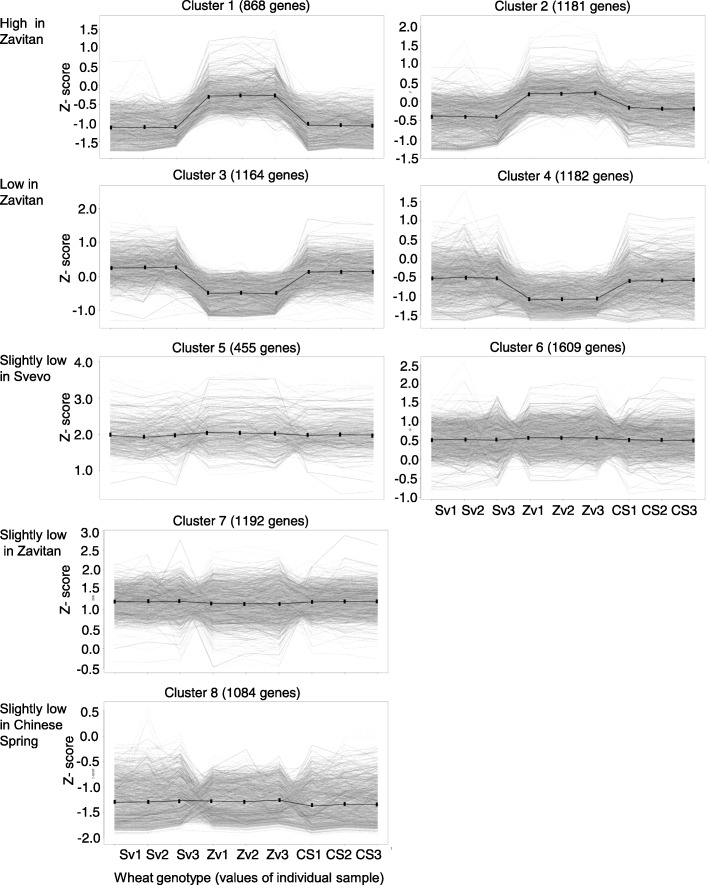
Gene expression patterns of the three wheat genotypes divided into eight clusters. A one-way ANOVA was performed on the transcriptomic dataset of the three wheat genotypes, Svevo, Zavitan, and Chinese Spring, as 11-day-old seedlings (*n* = 3). The analysis revealed a total of 8735 unique transcripts with significant expression profile changes for at least one wheat genotype. The *k*-means analysis was performed on scaled and centered rlog values, and it is represented by the Z-score. The total number of transcripts in each cluster is indicated, and the data for individual genes are shown in light gray. The expression responses for each cluster are shown in black. The names of each individual sample are presented on the x-axis

In order to detect differentially expressed genes in the RNA-seq data, the number of sequences associated with each gene (counts) for each sample was used to statistically compare between- and within-condition variability, by a negative binomial generalized linear model, using the R package DESeq2. The DESeq2 output, compared using the likelihood ratio test (LRT), was subjected to an rlog transformation, and the resulting heatmap clearly divided the overall transcriptional profiles of the two cultivated genotypes (Svevo and Chinese Spring) from the wild genotype (Zavitan), presented in Additional file 2: Figure S2. The analysis of differentially expressed genes (*q*-value < 0.05; |log_2_ (fold change)| > = 1) resulted in 8735 unique transcripts. We estimated the cluster number of the results using clusGap [44], which suggested dividing the data into eight clusters. The *k*-means analysis was performed on scaled and centered rlog values, and each cluster is represented by the Z-score (standard score) of gene expression from the set of genes showing similar response patterns in the three wheat genotypes (Fig. 2). The eight clusters were divided into five expression groups, derived from trends observed in the standard scores: i) Clusters 1 and 2: genes with a higher level in Zavitan than in Svevo and Chinese Spring. Cluster 2 presents genes with a higher Z-score than Cluster 1. ii) Clusters 3 and 4: genes with a lower level in Zavitan than in Svevo and Chinese Spring. Cluster 4 presents genes with a lower Z-score than Cluster 3. iii) Clusters 5 and 6: genes with moderately lower levels in Svevo than in Zavitan and Chinese Spring. Cluster 5 presents genes with a higher Z-score than Cluster 6. iv) Cluster 7: genes that have lower levels in Zavitan than in Svevo and Chinese Spring. v) Cluster 8: genes that have lower levels in Chinese Spring than in Svevo and Zavitan (Fig. 2). The distribution of genes into the eight clusters is presented in Additional file 1: Table S3.

To elucidate the metabolic processes, an over-representation pathway enrichment analysis was performed on each cluster using MetGenMAP [45], using rice orthologues (LOC gene ID; Additional file 1: Table S4). Table 1 describes the significantly enriched pathways of each cluster. The pathways that were significantly enriched in Clusters 1 and 2 (high in Zavitan) were mainly associated with polyamine biosynthesis and sugar degradation. The genes related to threonine and homoserine biosynthesis, phenylpropanoid biosynthesis, cell structure biosynthesis (cellulose) phospholipases, and ascorbate biosynthesis were associated with low expression in Zavitan (Clusters 3 and 4). The pathways that were significantly enriched in Clusters 5 and 6 (slightly low in Svevo) were mainly associated with the isoprenoid phosphate pathway, glycine, and glycerol degradation, brassinosteroid, jasmonic acid (13-lipoxygenase; 13-LOX) and gibberellin phytohormone biosynthesis, N-acetylgalactosamine biosynthesis, TCA-, Calvin-, and γ-glutamyl cycles and sugar degradation. The pathways that were significantly enriched in Cluster 7 (slightly low in Zavitan) were mainly associated with glutathione, β-alanine, methionine, homocysteine, and cysteine biosynthesis, as well as gluconeogenesis, tyrosine degradation, phospholipid desaturation, and fatty acid β-oxidation. Additionally, pathways that were significantly enriched in Cluster 8 (slightly low in Chinese Spring) were mainly associated with phytohormone cytokinin biosynthesis. Together, these observations indicate a unique gene expression for each wheat genotype that involves diverse pathways from primary and secondary metabolites, phytohormones, oxidation state, and cell wall.

**Table 1:** Enrichment analysis of metabolic pathways significantly over-represented in the three wheat genotypes

Gene expression pattern	Cluster number	Pathway Description	*P* value
High in Zavitan	1	acyl-CoA thioesterase pathway	1.05E-02
		sucrose degradation	1.29E-02
		starch degradation	1.87E-02
		β-D-glucuronide degradation	4.19E-02
	2	superpathway of polyamine biosynthesis	2.06E-02
		β-alanine betaine biosynthesis	3.56E-02
Low in Zavitan	3	threonine biosynthesis from homoserine	2.44E-02
		flavonoid biosynthesis	2.73E-02
		phenylpropanoid biosynthesis	3.37E-02
		de novo biosynthesis of pyrimidine deoxyribonucleotides	4.06E-02
	4	cellulose biosynthesis	2.70E-03
		trehalose degradation (high osmolarity)	1.47E-02
		phospholipases	1.76E-02
		ascorbate biosynthesis	2.24E-02
Slightly low in Svevo	5	isopentenyl diphosphate biosynthesis	3.52E-04
		glycine cleavage complex	7.60E-03
		divinyl ether biosynthesis II (13-LOX)	1.41E-02
		13-LOX and 13-HPL pathway	1.41E-02
		brassinosteroid biosynthesis	2.62E-02
		glycerol degradation	3.45E-02
		reductive TCA cycle	4.88E-02
	6	brassinosteroid biosynthesis	1.29E-03
		UDP-N-acetylgalactosamine biosynthesis	3.91E-03
		UDP-glucose conversion	4.78E-03
		Calvin cycle	4.78E-03
		GDP-L-fucose biosynthesis I (from GDP-D-mannose)	5.53E-03
		NAD salvage pathway II	9.16E-03
		UDP-galactose biosynthesis	1.10E-02
		ent-kaurene biosynthesis	1.17E-02
		dTDP-L-rhamnose biosynthesis	1.95E-02
		superpathway of GA12 biosynthesis	2.10E-02
		galactose degradation	2.22E-02
		γ-glutamyl cycle	2.55E-02
		GDP-D-rhamnose biosynthesis	4.59E-02
Slightly low in Zavitan	7	glutathione biosynthesis	1.91E-04
		β-alanine biosynthesis	3.66E-03
		gluconeogenesis	5.68E-03
		tyrosine degradation	5.82E-03
		phospholipid desaturation	1.87E-02
		fatty acid β-oxidation	3.98E-02
		glycolipid biosynthesis	4.11E-02
		methionine biosynthesis	4.11E-02
		homocysteine and cysteine biosynthesis	4.77E-02
Slightly low in Chinese Spring	8	cytokinins biosynthesis	1.48E-03

Gene expression patterns were sorted into eight clusters, as determined by a k-means analysis of transcripts detected in the three wheat genotypes, and analyzed using a MetGenMAP pathway enrichment analysis [45]

To determine which gene ontology categories were represented, we conducted a Singular Enrichment Analysis (SEA) using agriGO v2 [46], with default parameters. The International Wheat Genome Sequencing Consortium (IWGSC) database gene IDs were used as background in the SEA. In Additional file 1: Table S5, the GO terms of the biological processes of all eight clusters are presented in pairs. In Cluster 5 for example, the comparison between Zavitan-Svevo and Zavitan-Chinese Spring revealed biological processes related to isoprenoid biosynthesis, lipid metabolism, oxidation-reduction process, photosynthesis, and tetrapyrrole metabolism. The results of Cluster 5 are partially redundant with the pathway enrichment results (Table 1), regarding the lipid/13-LOX biosynthesis and the isoprenoid biosynthesis. The functional enrichment terms in this cluster are the most consistent with the differences in defense adaptations between the three genotypes. Other clusters included functions such as the organic acid metabolic process, the cellular amino acid metabolic process, cell redox homeostasis and metal ion transport (Cluster 7), the oxidation-reduction process (only differentially expressed genes of Svevo vs. Chinese Spring; Cluster 6), and the phenylalanine catabolic process (only differentially expressed genes of Svevo vs. Zavitan; Cluster 3). The remaining clusters included functional enrichment terms, such as protein phosphorylation, lipid transport, and recognition of pollen.

### Characterization of metabolic and physiological differences between the three wheat genotypes

Several pathways related to central metabolism, such as carbohydrate, amino, and fatty acids and the TCA cycle, showed variation in gene expression between the three genotypes (Table 1). Therefore, we performed a metabolic analysis of 11-day-old wheat seedlings using gas chromatography-mass spectrometry (GC-MS). This analysis revealed the relative levels of 72 metabolites, including amino acids, organic acids, sugars, and sugar alcohols (Additional file 1: Table S6). Table 2 shows the relative levels of 24 significantly different metabolites using a one-way-ANOVA. The results indicated that three aromatic amino acids, organic acids, and sugars were significantly lower in Svevo. Other metabolites showed no significant differences and are presented in Additional file 1: Table S6.

**Table 2:** Analysis of the central metabolites in the three wheat genotypes detected by gas chromatography-mass spectrometry (GC-MS)

		Svevo				Zavitan				Chinese Spring				one way ANOVA	
Class	Metabolite (relative abundance of the ion counts)	Average +/− SE				Average +/− SE				Average +/− SE				*F* value	*P* value (FDR)
Amino acid	Aspartic acid	9.41	+/−	0.48	ab	20.23	+/−	7.60	a	5.67	+/−	0.87	b	6.1	5.0E-02
	Phenylalanine	0.70	+/−	0.07	b	2.13	+/−	0.77	a	0.59	+/−	0.05	b	7.6	3.5E-02
	Tryptophan	0.01	+/−	0.00	b	0.05	+/−	0.02	a	0.02	+/−	0.01	ab	8.4	3.2E-02
	Tyrosine	0.02	+/−	0.01	b	0.10	+/−	0.03	ab	0.14	+/−	0.04	a	6.2	4.8E-02
Organic acid	Citric acid	5.40	+/−	0.32	b	17.03	+/−	6.09	a	4.73	+/−	0.46	b	8.1	3.3E-02
	Fumaric acid	0.28	+/−	0.02	b	1.03	+/−	0.28	a	0.41	+/−	0.06	b	11.7	1.4E-02
	Gluconic acid	0.14	+/−	0.01	b	0.99	+/−	0.39	a	0.30	+/−	0.02	b	9.0	3.0E-02
	Glyceric acid	1.92	+/−	0.11	b	5.57	+/−	1.88	a	4.46	+/−	0.57	ab	6.3	4.8E-02
	Hexadecanoic acid	3.95	+/−	0.35	b	12.22	+/−	2.09	a	5.98	+/−	1.24	b	12.6	1.3E-02
	Hydroquinone	0.15	+/−	0.01	b	0.38	+/−	0.11	a	0.14	+/−	0.02	b	8.3	3.2E-02
	Malic acid	5.51	+/−	0.52	b	18.82	+/−	4.77	a	7.15	+/−	0.71	b	13.5	1.2E-02
	Octadecanoic acid	4.92	+/−	0.39	b	14.30	+/−	2.28	a	7.23	+/−	0.91	b	18.7	7.2E-03
	Quinic acid	4.33	+/−	0.52	b	13.22	+/−	3.16	a	8.75	+/−	0.93	ab	10.6	1.9E-02
	Saccharic acid	0.72	+/−	0.06	b	2.23	+/−	0.75	a	0.13	+/−	0.01	b	13.4	1.2E-02
	Shikimic acid	4.11	+/−	0.73	b	7.43	+/−	2.33	ab	9.11	+/−	0.67	a	6.8	4.2E-02
	2-Oxogluconic acid	5.29	+/−	0.62	b	17.44	+/−	3.53	ab	23.68	+/−	5.90	a	6.7	4.2E-02
Sugar	Fructose	3.16	+/−	0.19	b	11.79	+/−	3.60	ab	20.36	+/−	5.05	a	7.5	3.5E-02
	Glucose	3.45	+/−	0.38	b	11.01	+/−	2.54	ab	16.29	+/−	2.56	a	14.4	1.2E-02
	Maltose	0.43	+/−	0.02	b	68.83	+/−	22.33	a	0.65	+/−	0.11	b	20.2	7.2E-03
	Ribose	0.56	+/−	0.05	b	1.75	+/−	0.60	a	2.39	+/−	0.15	a	19.0	7.2E-03
	Xylose	0.15	+/−	0.01	b	0.39	+/−	0.14	a	0.39	+/−	0.04	a	7.5	3.5E-02
	*N*-acetylmannosamine	0.37	+/−	0.02	b	1.06	+/−	0.38	a	0.99	+/−	0.04	a	8.8	3.0E-02
Sugar acid	Galactaric acid	0.24	+/−	0.01	b	0.56	+/−	0.18	a	0.03	+/−	0.01	b	14.2	1.2E-02
Sugar alcohol	Galactinol	1.93	+/−	0.21	b	10.73	+/−	3.63	a	1.90	+/−	0.35	b	12.1	1.4E-02

The metabolic profile was conducted in 11-day-old wheat leaves, and metabolites were normalized to the internal standard and presented as the relative abundance of the ion counts. The *P* value (fold discovery rate) was calculated using one-way ANOVA analysis (Tukey HSD), and the different superscript letters indicate statistical significance (mean ± SE, *n* = 3–6)

**Fig. 3:**
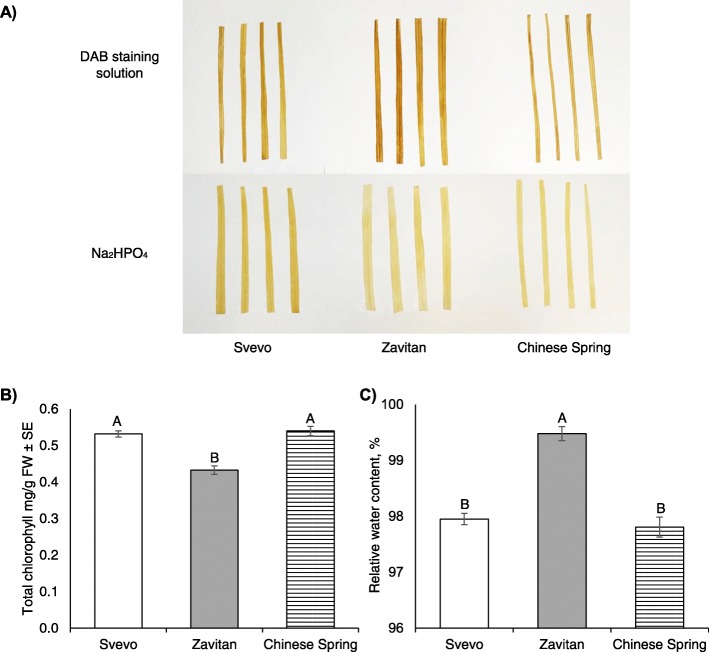
Physiological characterization of the three genotypes including ROS, total chlorophyll levels, and water content. **a** DAB staining of hydrogen peroxide levels in the wheat leaves. Upper panel: DAB solution; lower panel: Na_2_HPO4 solution, which was applied as a control treatment. **b** Total chlorophyll content in leaves of wheat plants (mean ± SE, *n* = 5). **c** The relative water content of three wheat cultivars (mean values ± SE, *n* = 5). Different letters above the bars indicate significant differences (*P* value < 0.05), one-way ANOVA using the Tukey-Kramer HSD test

Both the pathway enrichment analysis of Cluster 2 (Table 1) and the GO terms of Cluster 5 (Additional file 1: Table S5) revealed that genes related to polyamine biosynthesis and the oxidation-reduction process are differently expressed between the three genotypes. Polyamines serve as substrates for oxidation reactions that produce hydrogen peroxide (H_2_O_2_) both intra- and extracellularly [47]. One of the earliest signaling roles in many environmental stresses involves reactive oxygen species (ROS). As the most stable ROS, hydrogen peroxide plays a crucial role in physiological processes in plants, such as growth, development, and reproductive growth, and it is also involved in defense against pathogens and diseases [48]. We measured hydrogen peroxide in the leaves of 11-day-old plants using DAB staining (3,3′-Diaminobenzidine). The leaves of Zavitan generated more dark brown precipitate than the other two genotypes. The sodium phosphate control treatment showed no precipitate (Fig. 3a). This suggested that the oxidative status of Zavitan is higher than Svevo and Chinese Spring.

The effect of various abiotic and biotic stresses on the photosynthetic apparatus is inevitably associated with the formation of harmful ROS [49, 50]. In addition, both oxidation-reduction processes and photosynthetic processes were significantly represented in the GO term analysis (Additional file 1: Table S5, Cluster 5). As an indication of the differences in photosynthesis, we measured the total chlorophyll content by quantifying the levels of chlorophyll a and b [51]. As described in Fig. 3b, Zavitan leaves had significantly lower levels of total chlorophyll than Svevo and Chinese Spring leaves.

Genes related to sucrose and starch degradation were expressed at higher levels in Zavitan than in the other two genotypes (Cluster 1). Previous studies linked the effect of carbohydrate metabolism to the condition of the photosynthetic apparatus [52] and water content [53]. Therefore, we also measured the water content in the leaves, which is a critical indication of plant response to different environmental stresses [54–57]. As shown in Fig. 3c, Zavitan leaves have significantly higher water content than Svevo and Chinese Spring leaves. The leaves’ fresh, turgid, and dry weights are presented in Additional file 1: Table S7. Overall, these results support our transcriptomic analysis and show some similarities to the cultivated wheat genotypes relative to the wild emmer.

### Differences in gene expression related to chemical and physical defense adaptations

**Fig. 4:**
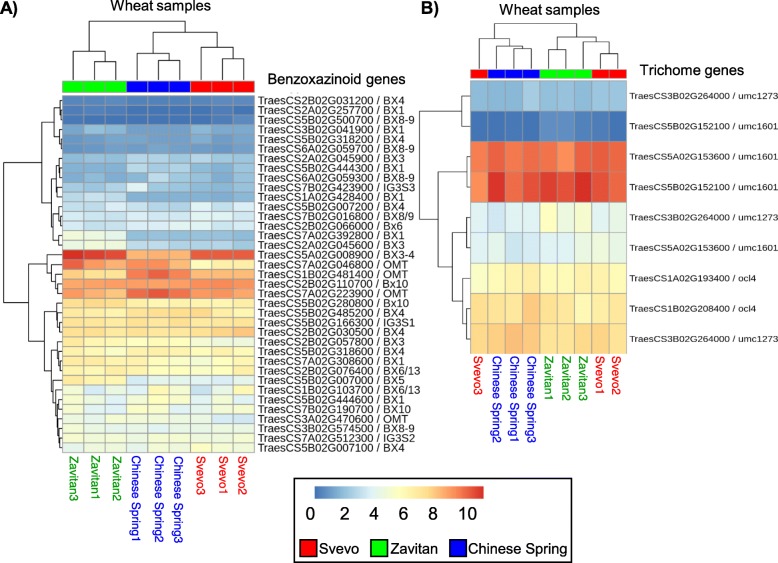
Heatmap of genes related to chemical and physical defense responses. Samples of the three wheat genotypes are sorted by hierarchical clustering (top). **a** Genes encoding proteins in the benzoxazinoid biosynthetic pathway. **b** Genes encoding to the benzoxazinoid biosynthetic pathway. Colors correspond with expression values; red indicates high expression levels, and blue indicates low expression levels

The pathway enrichment suggested that several pathways related to plant defense mechanisms differ between the genotypes. These include the 13-LOX and 13-HPL pathway, which is related to JA biosynthesis [58], isopentenyl diphosphate biosynthesis (terpenoids) [59], both in Cluster 5, and flavonoid biosynthesis [60] in Cluster 3 (Table 1). Therefore, we further investigated the gene expression of the benzoxazinoids as chemical toxic compounds and trichomes as a physical defense mechanism. To generate a gene list, we searched the BREADWHEATCYC2.0 database (www.plantcyc.org) and the literature (the benzoxazinoid biosynthetic genes named *Bx1* through *Bx14*) [16, 61, 62]. In the case of the unknown *Bx* genes in wheat (*Bx6, Bx7*, and *Bx10–14*), we aligned the maize protein sequences [63–65] to the wheat Ensemble Plant database (see Additional file 1: Table S8 for the full gene list). The expression values of *Bx* genes in each genotype are presented in the heatmap, and the samples are sorted by hierarchical clustering (Fig. 4a). The heatmap indicated that the samples of the cultivated wheat Svevo were closer to Chinese Spring than to Zavitan. It also showed that some genes, such as cytochrome P450, *Bx3,* and *Bx5,* were expressed at higher levels in Zavitan, while other genes, such as the downstream *glucosidases* and *O-methyltransferases*, were higher in Svevo and Chinese Spring. This suggested some similarities between Svevo and Chinese Spring regarding the benzoxazinoid biosynthesis genes.

We also compared the gene expression of trichome-formation-related genes as representative of a physical barrier [66, 67]. To generate a gene list, we searched the literature and found some evidence for trichome formation genes in rice, including *Glabrous Rice 1*, encoding a homeodomain protein [68], and the pubescence gene GL6 [69]. We also identified maize protein homologs, including i) HD-ZIP IV transcription factor OCL4, which is necessary for trichome patterning [70]; ii) UMC1273, which is a protein trichome birefringence-like 39; iii) UMC1601 protein trichome birefringence-like 28; iv) AY110056 protein trichome birefringence-like 26; and v) prm5 (powdery mildew resistant protein 5; (see Additional file 1: Table S9 for the full gene list). The expression values of the trichome formation genes in each genotype are presented in the heatmap, and the samples are sorted by hierarchical clustering (Fig. 4b). The heatmap did not show a clear pattern, as two Svevo samples were clustered with Zavitan and one was clustered with Chinese Spring.

### Quantification of changes in benzoxazinoid levels during plant development

**Fig. 5:**
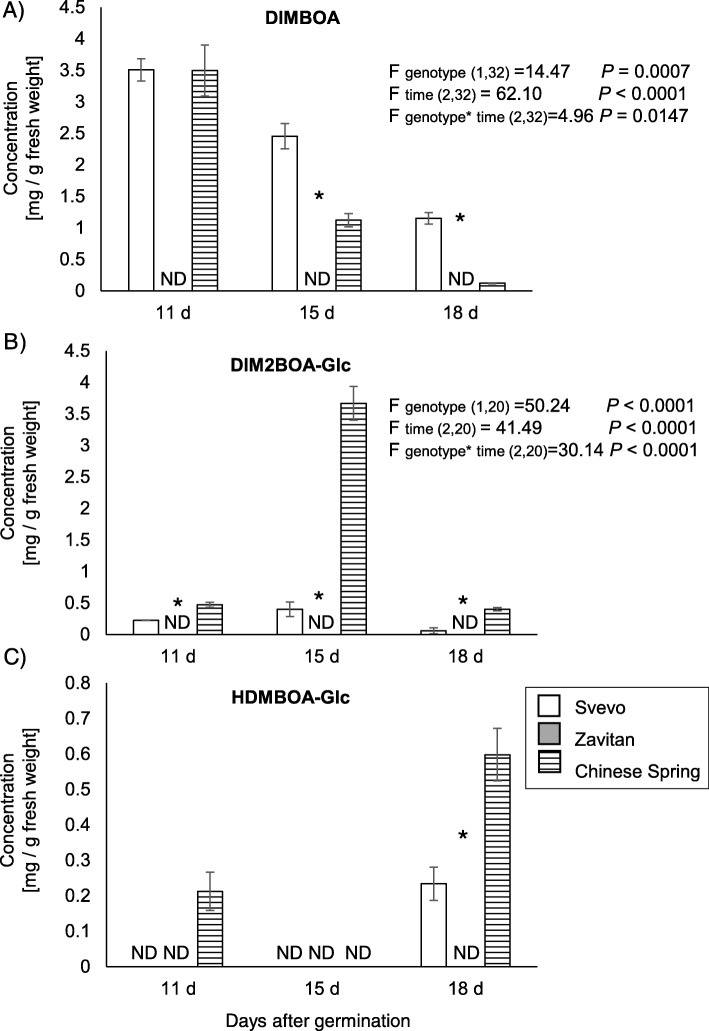
The benzoxazinoid levels in three selected wheat genotypes at 11, 15 and 18 days after germination. Leaves were harvested 11, 15, and 18 days after germination, and the samples were analyzed using HPLC-UV (mean ± SE, *n* = 2–6). A statistics Student’s t-test (*P* value < 0.05) was performed on the Svevo and Chinese Spring genotypes. **a** 2,4-dihydroxy-7-methoxy-1,4-benzoxazin-3-one (DIMBOA), **b** 2,4-dihydroxy-7,8-dimethoxy-1,4-benzoxazin-3-one glucoside (DIM_2_BOA-Glc), **c** 2-hydroxy-4,7-dimethoxy-1,4-benzoxazin-3-one glucoside (HDMBOA-Glc). The Student’s t-test (*P* value < 0.05) was performed at each time point, and the two-way analysis of variance (ANOVA) tested between genotypes and sampling time. ND: not detected

We focused our analysis of defense metabolites on benzoxazinoids (BXDs), which are major deterrent compounds and have been demonstrated to play a role in chemical defense in wheat leaves [71, 72]. The BXDs are abundant in wheat seedlings and show the highest activity in plants at the juvenile stage [14–16]. In this experiment, we measured, by HPLC-UV, the levels of BXDs in young wheat plants: 11, 15, and 18 days after germination. Overall, three BXD compounds were detected and identified, including 2,4-dihydroxy-7-methoxy-1,4-benzoxazin-3-one, 4-dihydroxy-7,8-dimethoxy-1,4-benzoxazin-3-one glucoside (DIM_2_BOA-Glc), and 2-hydroxy-4,7-dimethoxy-1,4-benzoxazin-3-one glucoside (HDMBOA-Glc) as presented in Fig. 5a-c. These compounds were further annotated by comparing the pattern of their fragment using UPLC-QToF-MS and previous studies (Additional file 1: Table S10 [73, 74];). All compounds were detected in at least one sampling time point, in the domesticated wheat genotypes, while they were below detection levels in the wild emmer Zavitan. The genotypes Svevo and Chinese Spring showed the highest levels of DIMBOA on day 11, which gradually declined until day 18. In Chinese Spring, DIMBOA levels declined more rapidly than in Svevo. DIM_2_BOA-Glc levels showed different accumulation patterns than DIMBOA, as the highest levels were detected on day 15 when Chinse Spring was much higher than Svevo. Overall, the two-way-ANOVA of both DIMBOA and DIM_2_BOA-Glc demonstrates significant differences in time (day after germination) and in the two wheat genotypes. The HDMBOA-Glc levels were much lower than the other two BXD compounds and only detected in Chinese Spring 11 and 18 days after germination and only at 18 days after germination in Svevo leaves.

### Calculating the trichome density

**Fig. 6:**
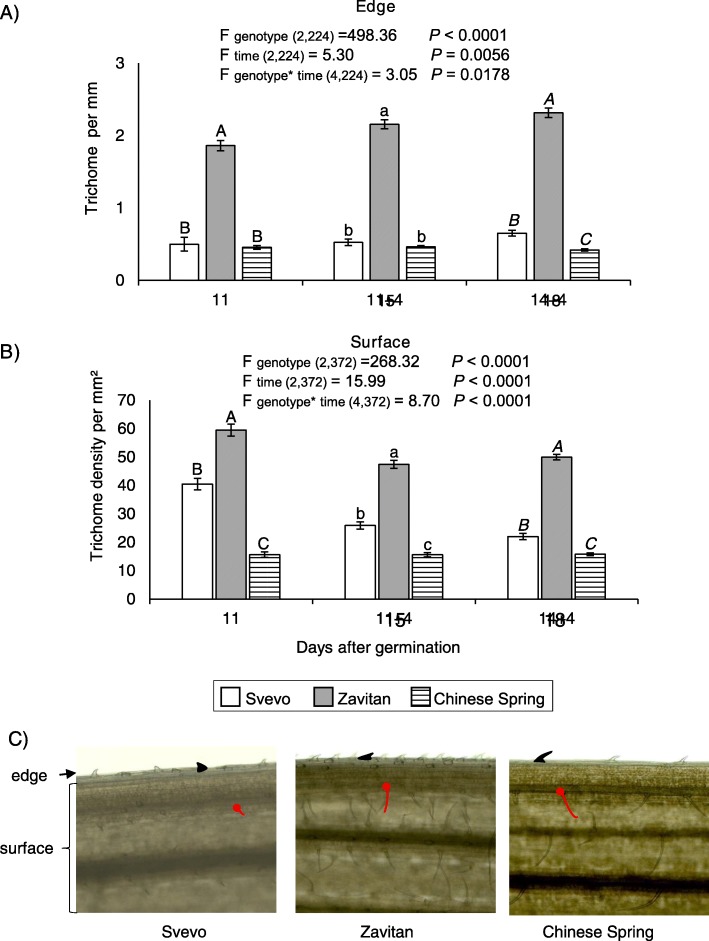
Trichome density on wheat seedlings at 11, 15 and 18 days after germination. Bars represent the average number of edge trichomes (**a**) and the density ± SE of surface trichomes (**b**) (mean +/− SE, *n* = 6–8). Different letters above the bars indicate significant differences, using ANOVA followed by Tukey’s HSD test separately for each sampling time. A summary table of the two-way ANOVA results is shown at the top. The inferior numbers indicate the degree of freedom and the total number of samples used for the test. **c** Images of leaf epidermis showing trichome length and direction (0.6 squared mm area of the leaf)

To explore the physical adaptation of wheat plants against aphid invasion, we evaluated the trichome density on the leaf edge and surface as a physical barrier (Fig. 6). As presented in Fig. 6a, the two-way-ANOVA suggests a significant difference in trichome number on the edges between the three wheat genotypes (F _genotype (2224)_ = 498.36, *P* value < 0.0001), the day after germination (F _time (2224)_ = 5.30, *P* value = 0.0056), and a cross-effect (F _genotype*time (2224)_ = 3.05, *P* value = 0.0178). The highest number of trichomes on the leaf edges was in Zavitan, while Svevo and Chinese Spring had a similar number of trichomes, except 18 days after germination, when Chinese Spring showed the lowest trichome number. The number of trichomes slightly increased over time, mainly on Zavitan and Svevo. In Fig. 6b, the trichome density on the leaf surface is presented. The two-way-ANOVA suggests a significant difference in trichome number on the surface between the three wheat genotypes (F _genotype (2372)_ = 268.32, *P* value < 0.0001), the day after germination (F _time (2372)_ = 15.99, *P* value < 0.0001), and a cross-effect (F _genotype*time (2372)_ = 8.70, *P* value < 0.0001). The highest number of trichomes on the leaf surface was in Zavitan, while Chinese Spring possessed the lowest trichome number. Images of the trichomes demonstrated that the trichomes are also different in their lengths and angles. As shown in Fig. 6c, the trichome lengths observed on the leaf surfaces of Zavitan and Chinese Spring were longer than those on Svevo. Additionally, the trichomes on the edges faced one direction only on Zavitan and Chinese Spring, while on Svevo, they faced both directions. Altogether, this suggested that Zavitan has more trichomes as a physical barrier than the other two genotypes.

### Evaluation of aphid reproduction on different wheat genotypes

**Fig. 7:**
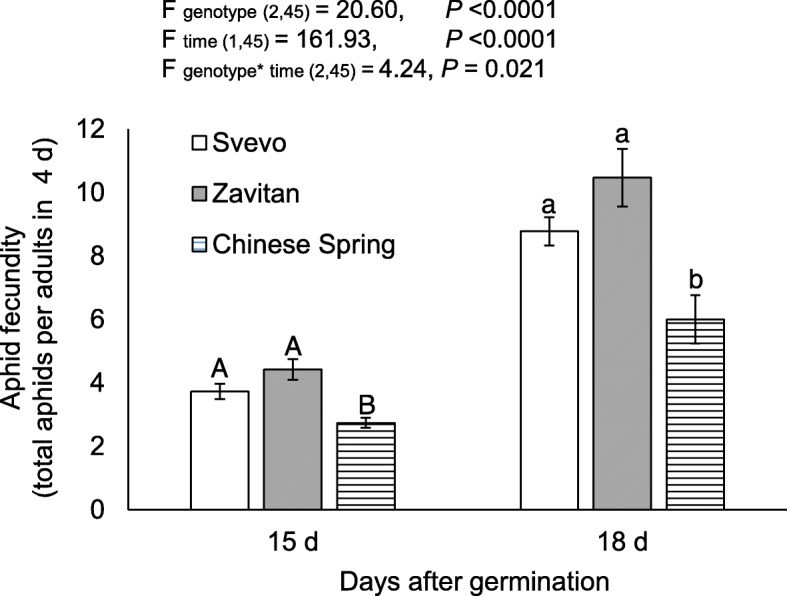
Aphid reproduction on three wheat genotypes at 15 and 18 days after germination. The total number of *R. padi* was counted after 96 h of infestation at two time points: 15 and 18 days after germination (mean ± SE, *n* = 5–10). The letters above the bars indicate significant differences between genotypes using a one-way analysis of variance (ANOVA) followed by Tukey’s HSD test (separate for each time point). A summary table of the two-way ANOVA results is shown at the top. The inferior numbers indicate the degree of freedom and the total number of samples used for the test

The aphid performance on wheat seedlings infested with aphids for 96 h was evaluated at two time points: 15 and 18 days after germination. The two-way ANOVA suggested a significant difference in the aphid progeny between the three wheat genotypes (F _genotype (2,45)_ = 20.60, *P* value < 0.0001), the day after germination (F _time (1,45)_ = 161.93, *P* value < 0.0001), and a cross-effect (F _genotype*time (2,45)_ = 4.24, *P* value = 0.021), indicating the effect of both genotype and age (day after germination) on aphid reproduction (Fig. 7). In the two measurements (15 and 18 days after germination), the Chinese Spring genotype was more aphid-resistant than the other two genotypes, while Zavitan and Svevo were not significantly different. Additionally, 18-day-old wheat seedlings were more susceptible to aphids than the 15-day-old plants.

## Discussion

In this research, we selected three wheat genotypes, including wild emmer, durum, and bread wheat, to address the fundamental question of the effect of domestication on plant resistance against aphids. To understand the overall gene levels and the differential gene expression between genotypes [75], we compared the transcriptomes of 11-day-old seedlings of the three genotypes. While the PCA plot based on the transcriptomic data suggested a unique pattern for each genotype (Fig. 1), the heatmap of the differentially expressed genes (8735 unique transcripts) indicated a higher similarity between the domesticated genotypes than the wild emmer wheat (Additional file 2: Figure S2). This pattern was similar when we compared the gene expression of specific biosynthetic genes of the benzoxazinoid pathway (Fig. 4). The similarity between Svevo and Chinese Spring is supported by a recent study that compared the exome of approximately 500 wheat genotypes and suggested that wild emmer wheat is the progenitor of the A and B subgenomes of all the modern tetraploid and hexaploid genotypes. The *T. durum* lineage was found to be the most likely ancestor of the bread wheat cultivated germplasm [20]. The similarity between Svevo and Chinese Spring might be due to a “domestication bottleneck” [21–23].

A previous study that explored the variation between 19 hexaploid bread wheat pangenomes reported that genes involved in the response to environmental stress and defense against biotic stress were variable between the genomes [76]. Similarly, in our results, several enriched functional genes involved in the response to environmental stress and defense responses are variable between the three genotypes, including polyamine biosynthesis and 13-lipoxygenase, which are the first enzymatic steps of the phytohormone, jasmonic acid (Table 1). Additionally, the over-representation pathway enrichment analysis demonstrated that genes associated with amino acid metabolism and the biosynthesis of carbohydrates, cell structures, fatty acids and lipids, phytohormones, and specialized metabolites were significantly different between the three genotypes. These observations indicated the major differences in the basal gene expressions between the genotypes. This was supported by several experiments where we measured the water content (Fig. 3c) and primary metabolites of 11-day-old leaves (Table 2). The main results suggested that Zavitan has higher levels of amino acids, organic acids, and sugars than the other two genotypes.

A recent study suggested that domesticated wheat has maintained its defense traits against specialized herbivores that have coexisted with the crop throughout its domestication, but that it is less efficient against generalist herbivores [31]. We compared the reproduction of the *R. padi* aphid on the three wheat genotypes (Fig. 7), which is among the most economically important aphid associated with a host range of well over 100 species [77, 78]. Our results show that the wild emmer, which possessed a high number of trichomes and had benzoxazinoid levels below detection, is more susceptible to the *R. padi* aphid than Chinese Spring (Figs. 5, 6 and 7). Zavitan was also more susceptible to the *R. maidis* aphid then Svevo [30]. In contrast to our results, a previous study by Migui and Lamb (2003) tested 41 accessions of wild and cultivated wheat in the field for resistance to three species of aphids, including *R. padi*. The results showed that the highest aphid resistance was in the diploid species and the lowest was in the hexaploid species [35]. This discordance may arise from accession-specific differences or may be due to the experiment being performed on mature wheat plants in field conditions, compared to young seedlings under controlled conditions (Additional file 2: Figure S1). This was supported by another study that suggests that the differences in aphid preference depend on the plant’s developmental stage in the field, compared to seedlings in the laboratory [79].

In wheat, genes encoding defense mechanisms are found in hexaploid bread wheat (genome BBAADD), tetraploid wheat (genome BBAA), and in the three diploid progenitors of hexaploid wheat (genomes AA, BB, and DD) [16, 80], while several of the enzyme steps are not yet identified. A comparison between the transcript levels from the three subgenomes in hexaploid wheat indicated that the homoeologs on the B subgenome are the main contributors to the benzoxazinoid biosynthesis pathway, especially in shoots [16, 81]. It was suggested that gene expression in diploids and tetraploids are far more varied than in the hexaploid population, indicating that the former species likely encode a greater allelic variation that, in turn, could facilitate breeding for pest resistance [82]. In this research, *Bx* gene expression was detected in all three genotypes, while the two domesticated wheat genotypes showed a closer pattern of gene expression and BXD levels than the wild emmer (Fig. 4a).

The BXDs are a diverse class of specialized metabolites, which are mainly known for their deterrent functions. They are known to have a crucial effect on plant resistance to insects such as aphids [71, 83, 84], chewing herbivores [85–87], fungal infection [88], and other insects, diseases and weeds [89]. A recent study reported that the molecular functions of the BXDs are diverse beyond their toxicity. It was suggested that the BXDs function in shaping the root microbiome by selectively attracting the plant-beneficial rhizobacterium *Pseudomonas putida* [90, 91]*,* and are also used as iron chelators [92]. Also, it has been estimated that naturally occurring DIMBOA may govern a physical defense mechanism against aphid feeding by the accumulation of callose [93]. Furthermore, it was reported that in maize, plant-aphid infestation caused secretion into the apoplast of DIMBOA-Glc, but not HDMBOA-Glc [71], which may indicate a unique function of DIMBOA-Glc. Our results indicated that the domesticated bread wheat Chinese Spring, which showed the lowest amount of aphid progeny, possesses a varied array of BXDs, including DIMBOA and DIM_2_BOA-Glc, and HDMBOA-Glc (Fig. 5).

**Fig. 8:**
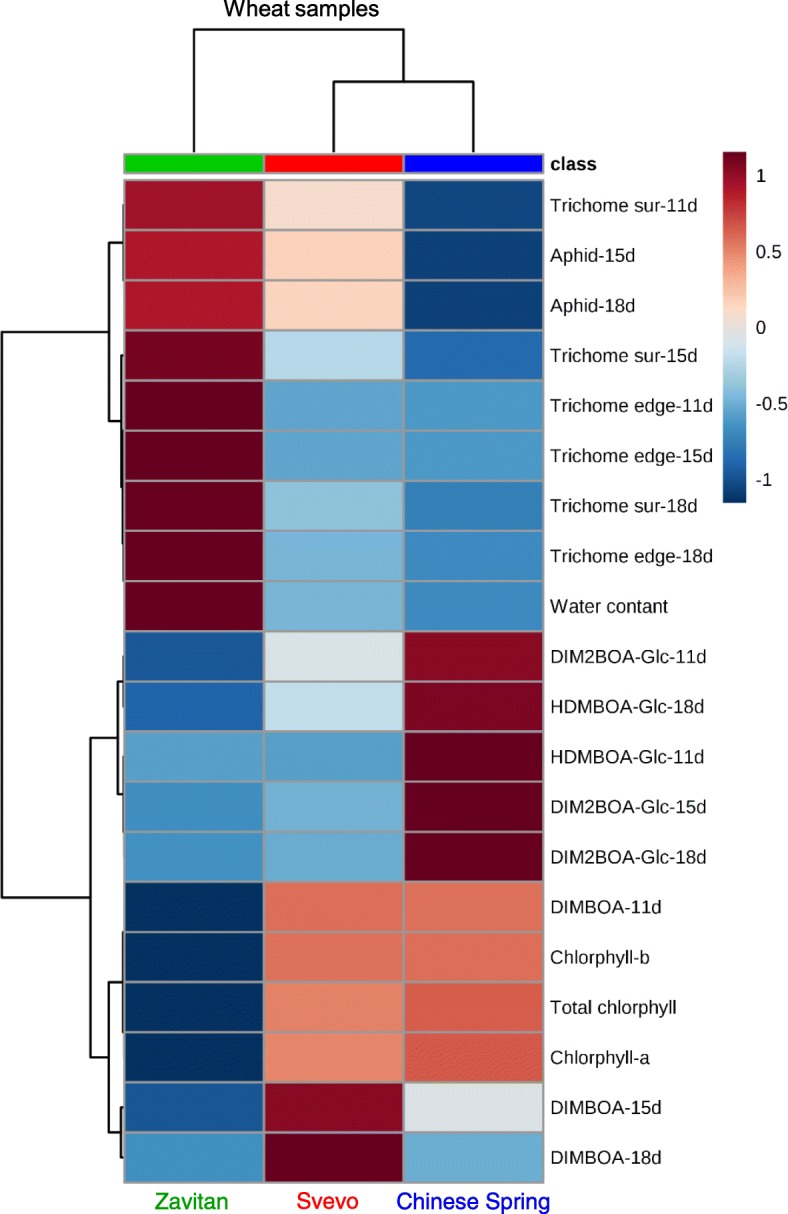
A summary heatmap of the physiological parameters and defense data used in this study. All the results were included besides the GC-MS primary metabolites due to a large amount of data. The Euclidean distance with Ward’s minimum variance method was designed using the default parameters of the MetaboAnalyst software. Colors correspond with concentration values (autoscale parameters); red indicates high levels, and blue indicates low levels

Interestingly, the total chlorophyll and chlorophyll a and b levels were clustered together with the amount of DIMBOA measured at 11 days (Fig. 8). Previous studies revealed a new function of BXDs as iron chelators in the roots [94, 95]. In plants, iron is an essential micronutrient that functions as a redox-active metal in many metabolic processes, including photosynthesis, mitochondrial respiration, nitrogen assimilation, hormone biosynthesis, and the production and scavenging of ROS [96]. Iron-deficient bread wheat plants exhibit significantly lower chlorophyll content and chlorosis, as well as low iron concentrations in leaves and grains [97]. Our results suggest that DIMBOA may play a role in iron-chelating in the leaves. Therefore, high levels of DIMBOA (as found in Svevo and Chinese Spring) may determine the chlorophyll levels or other processes that can affect photosynthesis. However, this requires further investigation.

BXDs are constitutively produced in young plants [14–16], and tend to decline as the plant ages [98]. Our results showed that aphid progeny numbers increased over time, while BXD levels decreased (Figs. 5, 7, and 8). The leaf surface is the first defense barrier against insects; it includes trichomes, thorns, silica, and wax [8, 99, 100]. Trichomes are used as a physical defense by disturbing herbivore movement, development, and oviposition, while glandular trichomes are used for exudate storage and secretion [101]. The surface density of trichomes and their dispersal on the leaf edges were not directly proportional to the number of aphid progeny. This suggests higher effectiveness of the chemical defenses compared with the physical defensive barrier. Additionally, the metabolic profile and water content indicated that 11-day-old Zavitan seedlings had higher water content, as well as higher levels of some amino acids, organic acids, and sugars, than the cultivated wheat genotypes. This lack of chemical defense and better water and nutrient status may be beneficial for aphid reproduction on Zavitan relative to the domesticated wheat.

## Conclusions

In this study, we combined transcriptomic, metabolomic and physiologic approaches to better understand the differences in wheat defense mechanisms. Our results suggest that benzoxazinoids provide a better defense mechanism than trichomes against *R. padi* aphids. Comparisons of significant gene expression, phenotypic characterization, and the chemical defense and physical responses indicated a higher similarity between the domesticated wheat genotypes than between either of them and the wild emmer. This suggested that under insect pressure, wheat plants might have undergone evolutionary convergence, which resulted in similarities in defense mechanisms via the biosynthesis of defense metabolites and, to a lesser extent, trichome formation.

## Methods

### Wheat genotypes

For this study, three wheat genotypes were selected: two tetraploids (*Triticum turgidum*) and a hexaploid (*Triticum aestivum*). The tetraploid wheat genotypes included: wild emmer wheat Zavitan (*Triticum turgidum ssp. dicoccoides*) [102, 103] and the durum wheat of Italian origin, Svevo (*Triticum turgidum ssp. durum*) [103, 104]. The hexaploid wheat was genotype Chinese Spring, which is widely used as a standard for wheat cytogenetic research [105, 106]. All plant material has been characterized and provided by Prof. Assaf Distelfeld (Tel Aviv University, Israel).

### Plant growth conditions

Wheat seedlings were grown on moistened HR2 (soil mix). Wheat seeds were planted 1.5–2 cm deep in 330-cm^3^ individual plastic pots and placed in a growth room. The growth room was maintained under a controlled light regime of a 16-h-light and 8-h-night photoperiod at a constant room temperature of 26 °C, a relative humidity of 63%, and a 250–350 μmol photon m^-2^ s^-1^ light intensity from a 3000 lm LED (LG-06A-12-364,000 k) at a distance of 40 cm from the light source (measured from the top of the plastic pot).

### Aphid bioassays

Bird cherry-oat aphids *(Rhopalosiphum padi)* were collected in the field and identified, and the colony was maintained on two-week-old wheat plants, a *Triticum aestivum* variety named Rotem (Agridera Seeds & Agriculture LTD, Israel). The colony was grown under a 16-h-light/8-h-dark photoperiod at a constant room temperature of 25 °C. For the insect bioassay, ten adult *R. padi* aphids (approximately 7–10 days old) were confined to the upper part of the second leaf of a seedling (11- and 14-day-old wheat) using clip cages (4.5 cm in diameter). To reduce variation in aphid age (which may affect reproduction), we applied ten aphids to each cage. Aphid progeny was calculated by counting the total number of aphids (adults and nymphs) after 96 h of infestation and dividing by ten (the number of adult aphids used in the experiment).

### RNA extraction, transcriptome sequencing, and analysis

Leaf samples were collected from the top of the second leaf. Tissue samples from two 11-day-old plants were combined into one experimental replicate, and three replicates were collected for each genotype. Total RNA was extracted using an SV Total RNA Isolation Kit with on-column DNaseI treatment (QIAGEN). The purified RNA was quantified, and 2.5 μg of each sample was used for next-generation sequencing using an Illumina HiSeq 4000 instrument with a 150 bp paired-end read length conducted by the GeneWIZ Company (www.genewiz.com). Quality control was performed using FASTQC and adapters, and low-quality sequences were trimmed and removed using Trimmomatic v0.36. Mapping was performed using a STAR aligner v2.5.2b against the *Triticum aestivum* reference genome v1.1 [38]. Reads aligning to exons were retrieved using Subread v1.5.2. For differential gene analysis, DESeq2 v1.22.2 [107] was used to perform a likelihood ratio test (LRT) to evaluate multiple genotypes at once (adjusted *p*-value < 0.05). The IWGSC refseq v1.0 high confidence (HC) functional annotation was used without sequences from the D genome, as this genome is not present in the wild and cultivated tetraploid wheat, and from the unknown genome. Next, DESeq2 was performed on the statistically significant genes from the LRT, and a regularized log was applied to the results. MetGenMAP was used to perform a pathway enrichment analysis [45], using rice ortholog IDs, which were converted from Phytozome wheat transcript IDs. In order to convert between IWGSC and Phytozome wheat transcript IDs, a reciprocal BLASTp comparison was performed, and only transcript IDs with mutual hits were retrieved. The MetGenMAP analysis allowed us to associate specific biochemical pathways with the differentially expressed genes found in our study. AgriGO v2 was used to perform a functional enrichment analysis of differentially expressed genes. This analysis identifies enriched gene ontology (GO) terms by comparing a query list of gene identifiers and their corresponding GO terms, with a background population list from which the query list was derived. The background list of genes and GO annotations was extracted from the International Wheat Genome Sequencing Consortium (IWGSC) database. The IWGSC is an international collaboration of 2400 members, which has produced a high-quality sequencing and annotation of the *T. aestivum* genome [108, 109].

### Benzoxazinoid extraction, analysis, and identification

Wheat tissue was collected from the top of the second leaf and ground to a fine powder under liquid nitrogen. Then, the frozen powder was weighed, and the 1:10 (w:v) ratio of extraction solvent contained 80% methanol (0.1% formic acid). The mix was vortexed briefly; then, the tubes were shaken for 40 min at 4 °C and centrifuged for 5 min at 14,000 g. Filtration was performed by centrifuging the samples on a 0.22-μm filter plate (EMD Millipore Corp., Billerica, MA, USA) at 3000 g for 5 min [74, 110, 111]. The extracted mixtures were covered with a WebSeal Mat and kept at 10 °C. Samples were injected into a DIONEX UltiMate 3000 high-performance liquid chromatography (HPLC) system using a C18 reverse-phase Hypersil GOLD column (3 μm pore size, 150 × 4.60 mm; Thermo Fisher Scientific, Germany). The column oven temperature was 40 °C and the UV-VIS absorbance spectra was at 190–400 nm. For BXD metabolite separation, a gradient of water (0.1% formic acid) (solvent A) and acetonitrile (0.1% formic acid) (solvent B) with a flow rate of 1 ml × min^− 1^ was used. The following linear gradient was used: 0 to 10 min, gradient from 5 to 55% B; 10 to 13 min, gradient from 55 to 100% B; 13–14 min, gradient from 100 to 5%; and 14–18 min, 5% solvent B. Chromeleon software (Thermo Fisher Scientific Inc.) version 7.2 was used for system control and data acquisition. For benzoxazinoid quantification, we compared the chromatograms with the authentic standards and plant crude extract. DIMBOA and DIBOA commercial authentic standards were used (Toronto Research Chemicals, Toronto, Canada). In addition, two crude extracts were used: i) a mix of DIMBOA-Glc:DIM_2_BOA-Glc in a ratio of 81:19, respectively, and ii) a mix of HDMBOA-Glc:HDM_2_BOA-Glc in a ratio of 86:14, respectively. Calibration curves were calculated by running authentic standards and crude extracts in different concentrations ranging from 0.5–50 μg/ml. The peak area of each compound was measured using Chromeleon software, and the final concentration was normalized to mg per gram fresh weight. Only three BXDs were detected in this analysis, namely DIMBOA, DIM_2_BOA-Glc, and HDMBOA-Glc. The UV spectra of the three BXDs are presented in Additional file 2: Figure S3 and Additional file 1: Table S11.

For accurate mass identification of the BXDs, 5 μl of 10 μg/ml authentic standards and crude extracts were injected onto an ultra-performance liquid chromatography-quadrupole time-of-flight mass spectrometer (UPLC-QToF-MS) system equipped with an ESI interface (Waters MS Technologies, Manchester, UK), ran in negative and positive ion modes. Chromatographic separation was carried out on a C18 column (100 mm × 2.1 mm, 1.7 μm), while the column was maintained at 40 °C, and autosampler was maintained at 10 °C. For lock mass calibration, analyses were performed using leucine enkephalin at a concentration of 0.4 ng/L, dissolved in 50% acetonitrile and 0.1% formic acid. The MS conditions were set essentially as described previously [112]. The sample ran in a gradient program including the mobile phase consisted of 95% water: 5% acetonitrile and 0.1% formic acid (solvent A), and 0.1% formic acid in acetonitrile (solvent B), in a flow rate of 0.5 ml/min, and the scans were repeated for 15 min in a single run as described previously [113]. For system control and data acquisition, MassLynx software (Waters) version 4.1 was used. For benzoxazinoid fragmentation patterns, we compared the chromatograms with the authentic standards of DIMBOA (Toronto Research Chemicals, Toronto, Canada), DIM_2_BOA-Glc, and HDMBOA-Glc from a plant crude extract and with previous publications [73, 74, 114]. The annotations of BXDs, DIMBOA, DIM_2_BOA-Glc and HDMBOA-Glc, and their fragmentation patterns are presented in Additional file 1: Table S10.

### Metabolic analysis using gas chromatography-mass spectrometry (GC-MS)

For metabolite extraction, 200 mg of ground frozen plant tissue was mixed with 1 ml of pre-cooled extraction solvents containing 55% methanol: 23% chloroform: 22% Milli-Q fileted water (Millipore, Merck), and 200 μl of 1 mg/ml ribitol and D-sorbitol ^13^C_6_ as internal standards. The samples were briefly vortexed, incubated in a thermomixer at 1000 rpm for 10 min at 25 °C, followed by 10 min of sonication; they were then centrifuged at maximum speed for 10 min, and the supernatant was collected. Next, 300 μl of chloroform and 300 μl of Milli-Q water were added, vigorously mixed, and centrifuged for 5 min at 6800 g. After phase separation, 100 μl of the top hydrophilic layer was collected and dried in a vacuum. Samples were derivatized by adding 40 μl of 20 mg/ml metoxyamine hydrochloride (Sigma-Aldrich) dissolved in pyridine following incubation for 2 h in an orbital shaker at 1000 rpm at 37 °C. Next, N-methyl-N-(trimethylsilyl) tri-fluoroacetamide (MSTFA), including a standard mix (Alkanes) in a volume of 77 μl, was added to each sample followed by incubation for 30 min in an orbital shaker at 37 °C. Samples were transferred to glass vials and loaded randomly onto a GC-MS single quadrupole mass spectrometer instrument (Agilent Technologies, Santa Clara, CA, USA). Then, 1 μl of the sample was injected into an inert flow path split/splitless inlet with glass wool (Restek, USA) in a 15:1 split ratio on a VF-5 ms capillary column (30 m long) with 0.25 mm i.d. and 0.25 μm film thickness + 10 m EZ-Guard (Agilent Technologies, Santa Clara, CA, USA). The Programmed Temperature Vaporisation (PTV) for injected samples ranged from 70 to 300 °C at 14.5 °C per sec; the transfer line was at 350 °C, and the ion source was adjusted to 250 °C with gain factor 1. Helium was used as a carrier gas with a constant flow rate of 1.8 ml per min. For primary metabolite analysis, the temperature program was as follows: 1 min isothermal heating at 70 °C, followed by a 1 °C/min oven temperature ramp to 76 °C, followed by a 6 °C/min oven temperature ramp to 340 °C, and a final 5 min heating at 340 °C. Mass spectra were recorded at 1.6 scans per second with a mass-to-charge ratio of 70 to 550 scanning range [115]. Data acquisition was conducted by Mass Hunter software and the NIST mass spectral library. Additionally, retention index (RI) libraries (Max-Planck Institute for Plant Physiology in Golm, (http://gmd.mpimp-golm.mpg.de/) were used for validation [113, 116]. Metabolites were normalized to D-sorbitol ^13^C_6_ as an internal standard and presented as the relative abundance of the ion counts.

### Trichome density

The upper parts of second leaves (same as those used for applying clip cages) were collected, and chlorophyll was bleached by 70% ethanol at 85 °C for 8 min, then rinsed with water. The tissue was placed on glass microscope slides facing to the adaxial side (leaf surface up). Trichome density images were photographed with a digital camera (Axiocam 305 color) connected to a Zeiss Axioplan 2 Upright Light Microscope (Zeiss, Oberkochen, Germany). For each leaf, nine photos were taken, including three from each side (left, right), and three from the middle (top, medium, and bottom positions). We counted the number of trichomes on the edges per mm and the density in mm^2^ using ImageJ software (https://imagej.nih.gov/ij/).

### Water content in the leaves

The second leaf (10 cm of tissue from the leaf tip) of each 11-day-old wheat seedling was collected. The total fresh weight was measured, and then the samples were placed in 2–3 ml of 5 mM CaCl_2_ solution for 8 h followed by drying in a 60 °C oven for 3–4 days; the dry weight was then measured. The calculation of the leaf relative water content was previously described [117]. The leaves’ fresh, turgid, and dry weights are presented in Additional file 1: Table S6.

### Detection of hydrogen peroxide

To examine the basal level of hydrogen peroxide in wheat leaves, a 3,3′-diaminobenzidine (DAB) staining was used for in situ detection [118]. In accordance with the aforementioned protocol, the second leaves of 11-day-old wheat seedlings were gently vacuum-infiltrated with DAB solution. As a control treatment, 10 mM of sodium phosphate was applied to replicate leaves. Following vacuuming, samples were incubated in a DAB solution for 4 h, which was then replaced with a bleaching solution (ethanol: acetic acid: glycerol 3:1:1) to remove the chlorophyll and to visualize the precipitate formed by hydrogen peroxide (which renders precipitates in dark brown).

### Chlorophyll content

Fresh leaf tissues (50 mg) were incubated in 5 ml of ice-cold 80% acetone for 48 h, centrifuged at 5000×g for 5 min, and absorbance was recorded at 663 and 645 nm wavelenghts. The amount of chlorophyll was calculated following the procedure of Arnon (1949) and expressed in mg g^− 1^ FW [51].

### Statistical analysis

For the principal component analysis (PCA) plot, the data were normalized using regularized log transformation, and the graph was plotted using the ggplot2 package in R. The one- and two-way ANOVAs (analysis of variance) used JMP software (SAS; www.jmp.com). In the two-way ANOVA, the inferior numbers for each *F* value indicated the degree of freedom and the total number of samples used for the test. Microsoft Excel was used for figure representation. In order to test the three genotype groups, a LRT was performed using DESeq2. LRT is a statistical test, similar to ANOVA, which allows the comparison of all levels of a factor at once. The number of clusters (*k*) was estimated using clusGap [44], and *k*-means clustering was performed with the k-means base function in R. The resulting gene clusters were evaluated for over- and under-representation with agriGO v2 [46].

## Supplementary information

**Additional file 1: Table S1.** Mapping sequence reads to the Chinese Spring reference genome. **Table S2.** Total RNA-seq values after rlog normalization. Annotations to the D subgenome or an unidentified subgenome (U) were eliminated. **Table S3.** Distribution of wheat genes into the eight clusters. **Table S4.** Gene annotation including the International Wheat Genome Sequencing Consortium database (IWGSC) and Phytozome gene ID. **Table S5.** Biological processes from the Singular Enrichment Analysis with agriGO v2 for significantly differentially expressed genes between each pair of genotypes. The data was divided into the eight *k*-means clusters. Only statistically significant GO terms are shown (FDR < 0.05). Queryitem: the number of genes containing the GO annotation; Querytotal: the total number of genes with GO annotations; bg item: the number of genes in wheat with this GO annotation; and bg total: the total number of genes in wheat with GO annotations. **Table S6.** Metabolites identified in leaves of 11-day-old wheat seedlings analyzed by GC-MS. The metabolites were normalized to the internal standard and presented as the relative abundance of the ion counts. **Table S7.** Weights of wheat leaf tissue used for water content calculation. The fresh, turgid and dry weights are measured in mg. **Table S8.** A full list of the *Bx* genes in bread wheat. The data include genes from Subgenome A, B, D, and U (not classified). **Table S9.** A full list of the trichome formation and regulation genes in bread wheat. The data include genes from Subgenome A, B, D, and U (not classified). **Table S10.** Benzoxazinoid annotation and fragment patterns detected and identified in wheat leaves by UPLC-QToF-MS analysis. **Table S11.** Levels of DIMBOA, DIM_2_BOA-Glc, and HDMBOA-Glc metabolites detected by HPLC-UV. Calibration curves were calculated by running authentic standards and crude extracts in different concentrations ranging from 0.5–50 μg/ml. The peak area of each compound was measured using Chromeleon software, and the final concentration was normalized to mg per gram fresh weight.

**Additional file 2: Figure S1.** Photos of the wheat genotypes used for this research over 11–18 days after germination. The plants possessed a similar phenology. **Figure S2.** Heatmap of differentially expressed genes from a likelihood ratio test (LRT) with the DESeq2 R package. The analytic output was subjected to rlog transformation. The heatmap presents hierarchical clustering of the different genotypes (horizontal axis) against hierarchical clustering of the differentially expressed genes (DEGs; vertical axis). The resulting heatmap clearly divided the overall transcriptional profiles of the two domesticated genotypes (Svevo and Chinese Spring) from the wild emmer (Zavitan). **Figure S3.** The UV spectra of known benzoxazinoids detected in wheat leaves using high-performance liquid chromatography (HPLC-UV).

## Data Availability

The datasets used and/or analyzed during the current study are available in this manuscript in the supplementary files or from the corresponding author on reasonable request.

## Abbreviations

BXD: benzoxazinoid compounds
DIM_2_BOA-Glc: 2,4-dihydroxy-7,8-dimethoxy-1,4- benzoxazin-3-one glucoside
DIMBOA: 2,4-dihydroxy-7-methoxy-1,4-benzoxazin-3-one
DIMBOA-Glc: 2,4-dihydroxy-7-methoxy-1,4-benzoxazin-3-one glucoside
HDMBOA: 2-hydroxy-4,7-dimethoxy-1,4-benzoxazin-3-one glucoside
