# Supplementary material for: Comparative transcriptomic and metabolic analysis of wild and domesticated wheat genotypes reveals differences in chemical and physical defense responses against aphids
Source: BMC Plant Biol. 2020 Jan 13;20:19. doi: 10.1186/s12870-019-2214-z (PMC6958765; doi:10.1186/s12870-019-2214-z)
Supplement: Supplementary file 2 — Additional file 2: Figure S1. Photos of the wheat genotypes used for this research over 11–18 days after germination. The plants possessed a similar phenology. Figure S2. Heatmap of differentially expressed genes from a likelihood ratio test (LRT) with the DESeq2 R package. The analytic output was subjected to rlog transformation. The heatmap presents hierarchical clustering of the different genotypes (horizontal axis) against hierarchical clustering of the differentially expressed genes (DEGs; vertical axis). The resulting heatmap clearly divided the overall transcriptional profiles of the two domesticated genotypes (Svevo and Chinese Spring) from the wild emmer (Zavitan). Figure S3. The UV spectra of known benzoxazinoids detected in wheat leaves using high-performance liquid chromatography (HPLC-UV). [file 12870_2019_2214_MOESM2_ESM.pptx]

## Slide 1
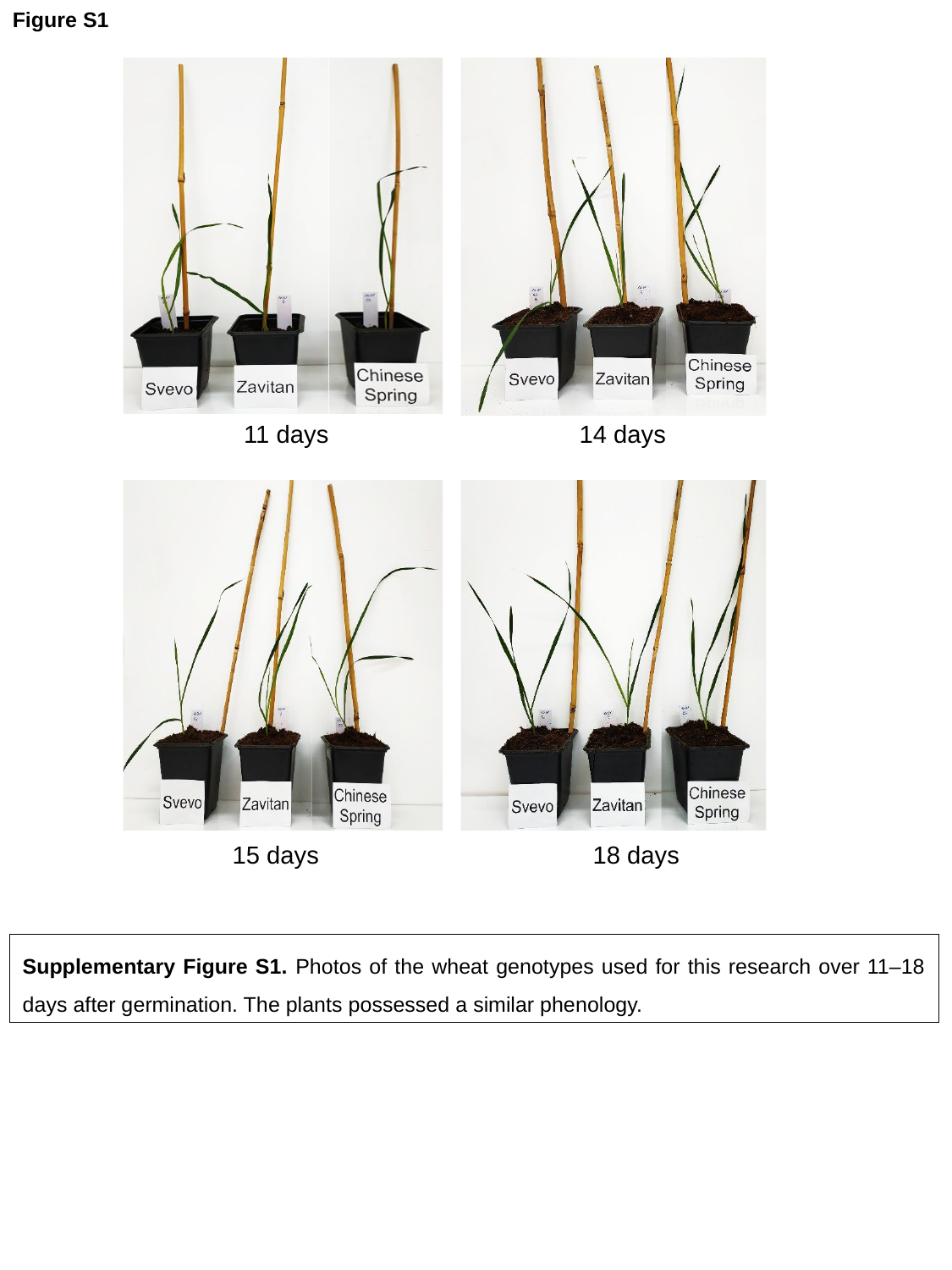

Figure S1
11 days
14 days
15 days
18 days
Supplementary Figure S1. Photos of the wheat genotypes used for this research over 11–18 days after germination. The plants possessed a similar phenology.

## Slide 2
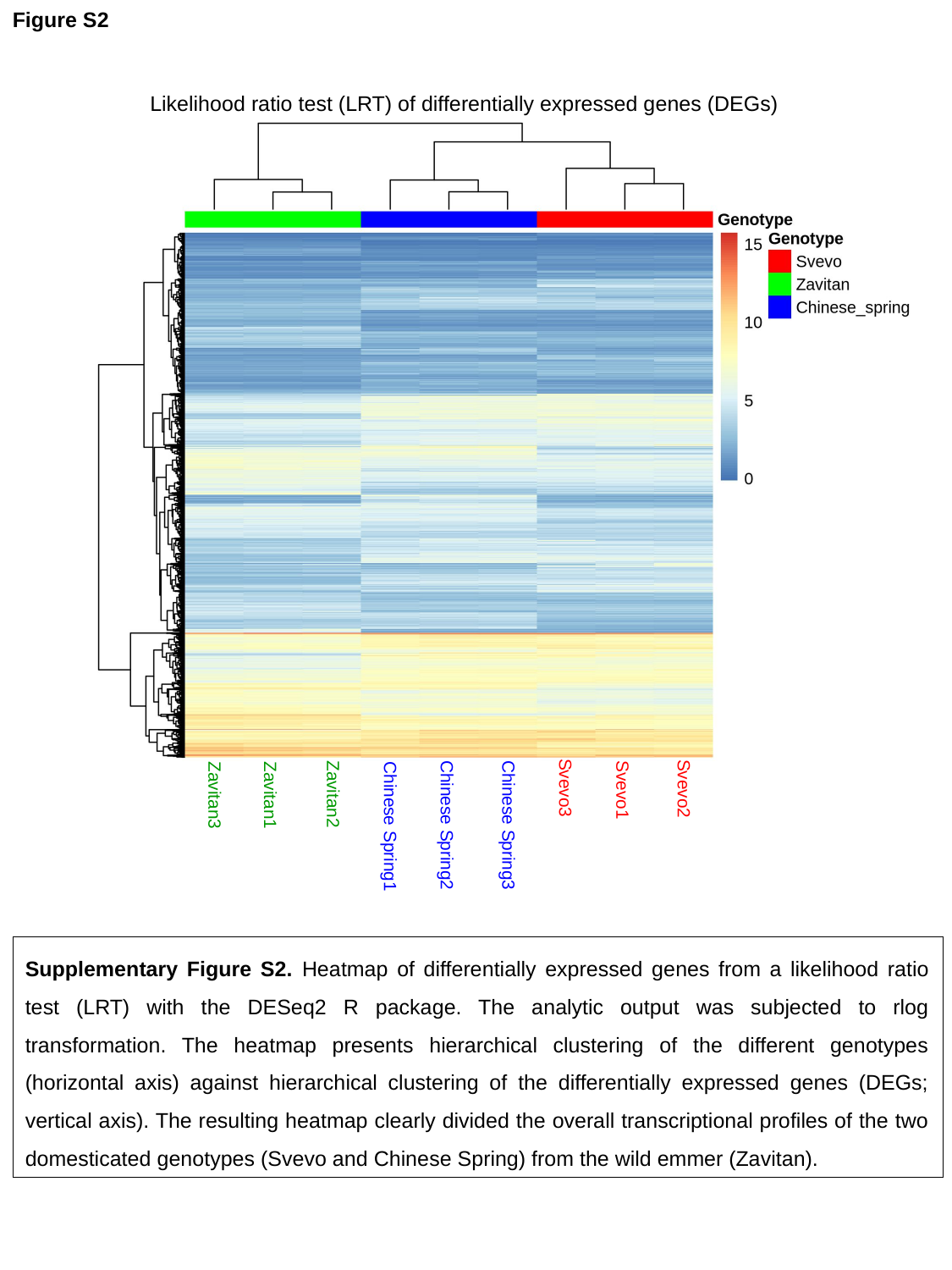

Figure S2
Likelihood ratio test (LRT) of differentially expressed genes (DEGs)
Zavitan2
Zavitan3
Zavitan1
Svevo3
Svevo2
Svevo1
Chinese Spring2
Chinese Spring3
Chinese Spring1
Supplementary Figure S2. Heatmap of differentially expressed genes from a likelihood ratio test (LRT) with the DESeq2 R package. The analytic output was subjected to rlog transformation. The heatmap presents hierarchical clustering of the different genotypes (horizontal axis) against hierarchical clustering of the differentially expressed genes (DEGs; vertical axis). The resulting heatmap clearly divided the overall transcriptional profiles of the two domesticated genotypes (Svevo and Chinese Spring) from the wild emmer (Zavitan).

## Slide 3
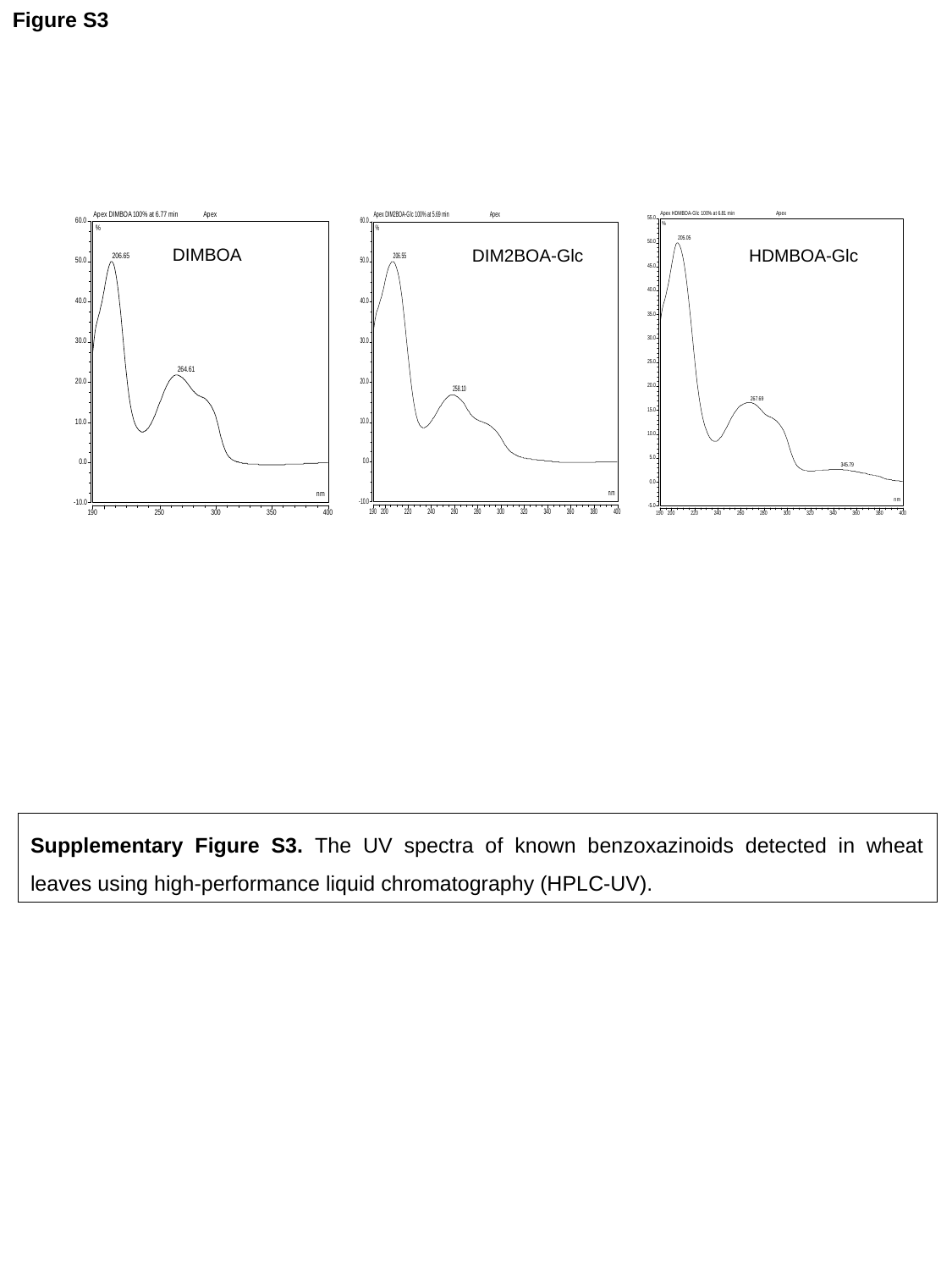

Figure S3
DIMBOA
DIM2BOA-Glc
HDMBOA-Glc
Supplementary Figure S3. The UV spectra of known benzoxazinoids detected in wheat leaves using high-performance liquid chromatography (HPLC-UV).
